# In dogs with osteoarthritis, is intra-articular allogenic mesenchymal stem cell therapy more effective than placebo effect?

**DOI:** 10.18849/ve.v7i3.473

**Published:** 2022-08-10

**Authors:** Megan Moloney+

**Keywords:** ADIPOSE DERIVED, ALLOGENIC, CANINE, LAMENESS, MESENCHYMAL STEM CELL, OSTEOARTHRITIS, PAIN, STEM CELL, UMBILICAL DERIVED

## Abstract

PICO question

In dogs diagnosed with osteoarthritis in the hip, elbow, stifle or shoulder joint, is treatment with intra-articular allogenic mesenchymal stem cell therapy, in comparison with a placebo effect, more effective at reducing lameness and pain?

Clinical bottom line

Category of research question

Treatment

The number and type of study designs reviewed

All three papers were randomised controlled trials

Strength of evidence

Weak

Outcomes reported

Intra-articular allogenic stem cell therapy is effective at reducing pain and lameness in dogs with osteoarthritis when compared to a placebo effect. Two studies indicated a statistically significant improvement in both client and veterinary outcome measurements. Client outcome measurements utilised included: the canine brief pain inventory; a measure of any changes in pain and lameness based on owners perception, and the client-specific outcome measure; and an evaluation of the impact of osteoarthritis on three client selected activities and how this changed with treatment. Veterinary outcome measurements included veterinary pain score based on manipulation of the limb, veterinary assessment of clinical outcomes and veterinary pre and post lameness examinations, all of which were subjective measures.

The final study identified a statistically significant improvement in both pain and lameness based on owner assessments utilising the canine brief pain inventory and the Hudson Visual Analogue Scale for lameness scoring. No statistically significant improvement was identified when considering subjective and objective veterinary measurements including force plate gait analysis and veterinary orthopaedic examination

Conclusion

There is moderate evidence from owner observation and veterinary assessment to suggest that intra-articular allogenic (adipose and umbilical derived) stem cell therapy has some efficacy for reducing pain and lameness compared to a placebo effect. However, it must be noted that these studies did not compare the use of intra-articular allogenic stem cells with conventional treatments such as intra-articular corticosteroid injections. Therefore, comparison trials are required.

Whilst all three papers showed significant improvements in the subjective measurements, objective data outcomes and assessment by board certified veterinary surgeons failed to find a significant improvement in peak vertebral force or lameness with the use of intra-articular stem cell therapy in comparison to a placebo effect. Furthermore, whilst no significant adverse reactions to intra-articular stem cell therapy were recorded, information regarding the safety for multiple dosing is lacking and ambiguity remains as to the most appropriate lineage and quantity of allogenic stem cells for the best clinical effect

How to apply this evidence in practice

The application of evidence into practice should take into account multiple factors, not limited to: individual clinical expertise, patient’s circumstances and owners’ values, country, location or clinic where you work, the individual case in front of you, the availability of therapies and resources.

Knowledge Summaries are a resource to help reinforce or inform decision making. They do not override the responsibility or judgement of the practitioner to do what is best for the animal in their care.

## The evidence

Two of the three papers (Harman et al., 2016; and Maki et al., 2020) investigated the use of allogenic adipose derived mesenchymal stem cells whilst the last paper (Kim et al., 2019) investigated the use of allogenic umbilical derived mesenchymal stem cells.

## Summary of the evidence

**Harman et al. (2016):** 

Population:	93 client owned dogs from nine different clinical study sites across the US.52 female and 41 male dogs varying from large to small breeds.Inclusion criteria: Age: 9 months + (average age was 8.1 years). Weight: 2.5 kg + (average weight was 29.63 kg). Signs and diagnosis: Physical exam and radiographic conformation of osteoarthritis (OA) in one or two of the following joints: hips, elbows, stifles or shoulders. Owner confirmed pain / lameness for at least 3 months prior and a subjective veterinary assessed pain on manipulation score of ≥ 3 based on a scoring system of 1–5 (where 1 indicated no response to palpation and 5 indicated the dog did not allow manipulation/palpation) for each arthritic joint. Exclusion criteria: Participants could not be pregnant, lactating or in oestrus. No known malignant or benign interfering neoplasia.
Sample size:	93 dogs.
Intervention details:	Adipose derived mesenchymal stem cells (MSCs) from a healthy donor dog were manufactured in accordance with standard operating procedures. Each study site was expected to enrol 10 participants, five for group A and five for group B. Overall 47 dogs were enrolled into group A and 46 into group B. Physical examination and radiographs were used to confirm the presence of OA. Participants were separated into two groups according to a blinded randomisation chart – group A treated with stem cells and group B treated with placebo. Dogs were sedated and given an intra-articular injection of either 0.7 ml of saline or 0.7 ml of stem cell solution that contained a target dose of 12 x 10^6^ viable adipose stem cells. Owners were instructed to restrict exercise post injection. Dogs were assessed at day 0 and day 60.
Study design:	Prospective, double-blinded, placebo controlled, randomised trial.
Outcome Studied:	Subjective: Client-specific outcome measurement (CSOM). Pain on manipulation – veterinary subjective pain on manipulation score based on patient response to manipulation of the limb. Veterinary assessment of clinical outcomes – veterinary global score. Owner assessment of clinical outcomes – owner global score. Safety – monitoring for adverse events.
Main Findings (relevant to PICO question):	Owner CSOM showed a statistically significant improvement in the treated versus the placebo group (p = 0.02). Veterinary pain score on manipulation showed a statistically significant improvement in the treated versus the placebo group (p = 0.01). Veterinary global outcome score showed a statistically significant improvement in the treated versus the placebo group (p = 0.0085). Owner global outcome showed an improvement in the treated versus the placebo group however this improvement was not statistically significant. 15 adverse events were reported throughout this trial: six in the treated group and nine in the control group. In each group, two of these events were deemed to be serious however all were assessed to be not related nor caused by the administered cell product. In total 74 of the original 93 enrolled dogs completed the study, 38 in group A (the stem cell treated group) and 36 in group B (the placebo group).
Limitations:	Uncontrolled environmental conditions – dogs were taken home with discharge instructions however owner compliance is uncontrollable. Loss of 19 participants to follow-up due to non-compliance with the protocol (11) and low enrollment at sites (eight). Difference in severity of OA between dogs – dogs were allowed to have one or two joints affected. Subjective outcomes. Measure of pain on manipulation of the limb by a veterinarian was subjective. Quantity of viable adipose stem cells unmeasured – potential for discrepancies between the amount of viable stem cells injected into each patient. Conflict of interest – not a truly independent study. The lead author is an employee and shareholder of the funding company, VetStem Biopharma.

**Kim et al. (2019):** 

Population:	51 client owned dogs (28 male and 23 female) at the University of Florida Small Animal Hospital. Inclusion criteria: Age: 12 months to 11 years. Weight: 11.5 kg to 60 kg. Signs and diagnosis; visible unilateral forelimb lameness for more than 6 months attributable to elbow osteoarthritis (OA). Osteoarthritic changes to the elbow confirmed on computerised tomography (CT) imaging. Owner assessment of Canine Brief Pain Inventory score (CBPI) (Hudson et al., 2004) greater than 2 for baseline pain severity and pain interference scores. General health; otherwise healthy. Any medication must have been at a stable dose 4 weeks prior to and throughout the trial. Exclusion criteria: Lameness for any reason other than forelimb elbow OA.
Sample size:	51 dogs.
Intervention details:	51 dogs were randomly assigned to umbilical cord derived mesenchymal stem cell (UMSC) therapy or placebo groups via the method of minimisation. Stem cells were obtained and cell banks generated from canine umbilical cords. All dogs were sedated and underwent arthrocentesis to confirm the presence of osteoarthritis in the joint via cytological evaluation of the joint fluid. 28 dogs were injected with intra-articular UMSC (0.5 ml containing approximately 7 x 10^6^ cells) and 23 with intra-articular saline (0.5 ml) via the preplaced needle used for arthrocentesis. Owners were advised to restrict their dog’s activity for 2–3 days post injection. All dogs were assessed at baseline, 1, 3 and 6 months post treatment using force plate gait analysis, owner assessed Hudson Visual Analog Scale (HVAS) and CBPI and veterinary orthopaedic exams.
Study design:	Prospective, double-blinded, placebo-controlled randomised trial.
Outcome Studied:	Objective: Force plate gait analysis. Subjective: Owner assessed CBPI level of pain – primary outcome measure. Owner assessed degree of lameness using HVAS. Veterinary orthopaedic exam. Safety – monitoring for adverse events.
Main Findings (relevant to PICO question):	No significant difference in age, body weight, proportion of dogs using non-steroidal anti-inflammatory drugs (NSAIDs) and symmetry index between the treatment and placebo groups. Owner assessed CBPI scores – statistically significantly higher treatment success rate in the UMSC treated group compared with the control group at 1 and 6 months after treatment. No significant difference (p = 0.056) observed at 3 months. Mean HVAS mood and sum indexed HVAS scores both significantly improved following treatment in the UMSC group. No significant differences were observed for the placebo group. Plate vertical force (PVF) – no differences were seen in either the treatment or control group. Veterinary orthopaedic exam – no differences seen in either group. Seven serious adverse events occurred – five in the UMSC group, and two in the placebo group. All were classified as unlikely to be related to treatment.
Limitations:	Lack of representation of the wider clinical population – the paper assessed the efficacy of UMSC treatment on unilateral osteoarthritis cases. The majority of osteoarthritis cases seen in dogs are bilateral. Under-powered study – the group sizes for the UMSC and placebo intervention were different and the total number of subjects completing the study was below the original target set by the pre-study power analysis. Subjective primary outcome variable CBPI - prone to bias. Bias was limited by blinding and subjecting both groups to the same confounding variables. Less stringent definition of success based on CBPI – a reduction of pain interference score (PIS) >2 is usually used as a measure of success whereas this study utilised a reduction of PIS >1 to indicate success. Uncontrolled environmental conditions – dogs were taken home with discharge instructions however owner compliance was uncontrollable. No indication as to whether the same veterinarian conducted the orthopaedic exam on the dogs at each time point. Only the subjective outcomes studied in this paper improved with the UMSC treatment, objective outcomes measured did not. Short time period – in total the study only ran for 6 months. Conflict of interest – several authors were employed by the Animal Cell Therapies company, though it is declared that the research was conducted in the absence of commercial relationships.

**Maki et al. (2020):** 

Population:	20 client owned dogs, 12 female and 8 male, from two private veterinary clinics in Hong Kong.Inclusion criteria: Age: 1 year + (median age 11.25 years). Weight: 9 kg + (median weight 25.5 kg) and body condition score of 7/9 or less. Signs and diagnosis: clinical signs of osteoarthritis (OA) for at least 1 month in one or both hip joints with radiographic evidence of arthritic changes. Noticeable lameness, limited range of motion, and evident pain on palpation / manipulation at the time of evaluation. Patient must have undergone at least 1 month of medical and / or physical therapy / cage rest management with little or no improvement and all treatments had to be stopped at least a week prior to start of trial. Exclusion criteria: Patients could have no additional significant illnesses nor have had any surgery in the affected area within the previous year.
Sample size:	20 dogs.
Intervention details:	Stem cells were obtained from fat tissue of a healthy 5 month old female donor dog during an ovariohysterectomy. All patients were put under general anaesthesia for treatment. 16 dogs were injected intra-articularly with mesenchymal stem cells (MSCs) at different concentrations – five received 5x10^6 ^cells per joint, six received 25x10^6 ^cells per joint, and five received 50x10^6 ^cells per joint. The other four dogs were injected intra-articularly with saline. Blood was taken and pain and lameness scores were recorded before treatment, at day 0 (day of treatment), day 5, day 30 and day 90 after the injection.
Study design:	Prospective, double blinded, placebo controlled, randomised control trial.
Outcome Studied:	Objective: Blood – anti / pro inflammatory and immunomodulatory biomarkers. Subjective: Lameness score – owner canine brief pain inventory (CBPI) score and veterinary pre and post assessment forms. Pain score – owner CBPI.
Main Findings (relevant to PICO question):	Lameness scores – 6/7 (86%) of the dogs with low to moderate lameness scores showed improved lameness scores following MSC administration however, only 3/4 (75%) of dogs with severe lameness scores showed improved lameness scores (P <0.05). Dogs injected with MSC had a statistically significant improvement of lameness within the first 30 days (P <0.05). Overall compared with the placebo group, improvement in lameness with MSCs was extremely statistically significant (P <0.0001). Interleukin receptor antagonist protein (IRAP) levels – all dogs that were seen to have improvement in lameness also had increased IRAP levels indicating that increased IRAP levels could be a good indicator for lameness improvement. (No power factor was given so to be taken with caution). CBPI owner assessed pain scores – results mirrored the veterinary assessed lameness scores of the participants. All animals receiving MSCs were seen to have improved pain scores. No statistical significance between age of dog and lameness response to MSC treatment or sex of the dog and lameness response to MSC treatment.
Limitations:	Small sample size and small placebo group containing only four participants – very underpowered study. Variable group sizes. Volume of stem cell injection and saline injection not stated ­– unknown whether they were the same or not. Varying degrees of OA in all participants. Subjective primary outcomes. Injection site ambiguity – entry into the joint was not always confirmed by aspiration and was sometimes only judged on ‘surgeon feel of a slight pop’. Increased chance for human error. Uncontrolled environmental conditions – owner instructions for at home care were broad and not monitored. Ambiguity in the numbers of enrolled dogs at each stage with attrition not clearly described. Conflict of interest – the study was funded by VetCell Therapeutics (VCT) USA and VCT Asia who are both stem cell manufacturers. Two dogs were lost to follow-up due to receiving other medications / treatment during the study period.

## Appraisal, application and reflection

The aim of osteoarthritis treatment is to decrease pain and increase limb function – enhancing the quality of life of the patient. The current treatment of choice for canine osteoarthritis (OA) is non-steroidal anti-inflammatory drugs (NSAIDs) alongside other conventional therapies including the use of polysulphated glycosaminoglycans, nutritional modifications, physical therapy and weight management (Pettitt & German, 2015). Although proven to be effective at combatting pain and lameness in dogs suffering with OA, owner compliance to daily administration of NSAIDs can be poor (Harman et al., 2016). New interventions such as platelet rich plasma (PRP) and mesenchymal stem cell injections are becoming increasingly popular for their reduced side effects and safety as long-term treatment (Singh, 2012). There are two forms of stem cell therapy available, allogenic and autologous. Most commercially available stem cell therapies for use in dogs are autologous. This Knowledge Summary looked into the efficacy of the use of allogenic stem cells for treatment of OA when compared to a placebo effect. The papers reviewed provided some moderate subjective evidence. No objective evidence was observed. One of the papers (Kim et al., 2019) showed no improvement in lameness based on both subjective and objective veterinary measurements thus indicating that allogenic stem cell treatment alone may have a limited effect on improvement of lameness.

Comparisons between the use of autologous and allogenic stem cells for OA in dogs are yet to be investigated. Both forms of stem cell therapy offer benefits and limitations. Allogenic stem cells offer the benefit of being available ‘off the shelf’ and appear to have a relatively long duration of action (Kim et al*.*, 2019) however questions as to the safety of multiple dosages of allogenic stem cells should be considered. Studies of allogenic stem cell use in animals for other disease processes have identified complications due to graft-versus-host disease (Michálek et al., 2003; and Wi et al*.*, 2021). All three papers reviewed only assessed single dose interventions and the longest time period studied in any of these papers was 6 months (Kim et al., 2019), meaning that long-term adverse effects and the total duration of improvement of a single intra-articular injection of stem cells is unknown. There is also consideration to be made as to the ethics of collecting allogenic stem cells. In two of the studies (Kim et al*.*, 2019; and Maki et al*.*, 2020) the stem cells were collected as a by-product from donor dogs that were already undergoing surgery for other procedures, however in the final study (Harman et al*., *2016) it is not stated as to whether the donor dog was undergoing a general anaesthetic for any reason other than stem cell collection. Collection of allogenic stem cells carries all the risks of a general anaesthetic and does not benefit the donor in any way thus it poses a major ethical issue. Autologous stem cells pose less of a risk when considering the patients’ immune system (Khaddour et al., 2020) however they require harvesting from the patient themselves which poses a risk through the need for surgical collection. Autologous stem cells also pose an ethical issue as their production warrants the need for at least two general anaesthetics for the patient (RVC Canine Stem Cell Treatments Owners' Frequently Asked Questions, 2021). Further safety data is required for both forms of stem cell therapy to determine the risk, benefit ratio.

Mesenchymal stem cells can be obtained from a number of different tissues in the body (Hass et al., 2011). Both adipose and umbilical derived stem cells were used in these studies. One study (Kim et al., 2019) suggested that umbilical derived stem cells may be better than adipose derived as they are a population of younger stem cells with a greater ability to differentiate and proliferate. As well as stem cell lineage, the number of viable stem cells in the solution injected into the osteoarthritic joints differed amongst all three papers. One paper (Maki et al., 2020) compared intra-articular injection of different quantities of stem cells and did not identify any significant difference between lower and higher dosages in any of the measured outcomes. Increased volumes of stem cell did appear to have an increased level of IL-10 biomarker in the patients’ serum however, due to sample sizes this correlation was not significant. Age, lineage and quantity of the stem cells may well impact their efficacy and therefore it may be beneficial for future studies to compare these.

The sample size across all three studies was small. Only one of the papers (Kim et al., 2019) had done a pilot study to calculate a power analysis and even in this study the clinical trial sample size was smaller than that calculated therefore indicating a lack of power in the study. Not having a large enough study group could mean that the results were not true of the wider population and statistical analysis done on the results would have been invalid (Suresh & Chandrashekara, 2012).

All studies were blinded.  The necessity for blinding was obvious in all three studies as there was a significant placebo effect noted in each. However, the effects of the stem cells went beyond that of the placebo.

The papers reviewed all used subjective primary measure outcomes which have a high potential for bias. This bias was mitigated through use of two well regarded subjective measures – Canine Brief Pain Inventory and Hudson Visual Analog Scale scores, both of which have been evaluated in multiple studies and are well accepted (Hudson et al., 2004; and Brown et al., 2008). Force plate gait analysis used in one paper (Kim et al*.*, 2019) is an objective measure however it is very difficult to take accurately and has lots of room for error (McLaughlin, 2001).

In all of the papers the animals were client owned and taken home throughout the duration of the study and therefore there was a lack of environmental control. However, this provided a perspective on how the animal coped within their usual environment.

There was a potential limitation in the conflict of interest in two of the papers (Harman et al*.*, 2016; and Kim et al*.*, 2019). Both papers had input from employees of major stem cell research companies. This limitation was mitigated through the declaration of the remaining authors expressing that the research was conducted in the absence of any commercial or financial relationship however further funding is required for true independent studies to take place.

Overall, there is weak evidence to suggest that allogenic stem cell use in dogs with osteoarthritis is more efficacious than a placebo effect. However, consideration must also be taken when providing allogenic stem cell use as a treatment to owners such as donor, recipient, dosing, cell therapy formulation, route of administration and veterinary surgeon experience (Maki et al., 2020). Whilst these studies have suggested that allogenic stem cell use is effective as treatment for canine OA when compared to a placebo effect, it would be more useful to compare and recognise its uses to the current known treatments for canine OA, such as NSAIDs or intra-articular corticosteroids.

## Methodology Section

**Search Strategy:** 

Databases searched and dates covered:	CAB Abstracts via CAB Direct (2007–2021)Scopus via Scopus.com (2005–2021)PubMed via the NCBI interface (2005–2022)
Search strategy:	CAB Abstracts: ((“stem cell*”) AND (OA OR osteoarthritis) AND (shoulder OR stifle OR hip OR elbow) AND (dog OR canine) AND (placebo OR control)) ((“stem cell*” OR mesenchymal OR MSC OR stem) AND (osteoarthritis OR OA OR DJD OR “degenerative joint disease”) AND (shoulder OR stifle OR hip OR elbow) AND (canine OR dog) AND (placebo OR control)) Scopus: ((“stem cell*”) AND (OA OR osteoarthritis) AND (shoulder OR stifle OR hip OR elbow) AND (dog OR canine) AND (placebo OR control)) ((“stem cell*” OR mesenchymal OR MSC OR stem) AND (osteoarthritis OR OA OR DJD OR “degenerative joint disease”) AND (shoulder OR stifle OR hip OR elbow) AND (canine OR dog) AND (placebo OR control)) PubMed: ((“stem cell*”) AND (OA OR osteoarthritis) AND (shoulder OR stifle OR hip OR elbow) AND (dog OR canine) AND (placebo OR control)) ((“stem cell*” OR mesenchymal OR MSC OR stem) AND (osteoarthritis OR OA OR DJD OR “degenerative joint disease”) AND (shoulder OR stifle OR hip OR elbow) AND (canine OR dog) AND (placebo OR control))
Dates searches performed:	06 Jan 2022

**Exclusion / Inclusion Criteria:** 

Exclusion:	Treatment group received additional therapies beyond allogenic stem cell therapy. Stem cells were obtained from a different species. Stem cells were not allogenic. Stem cells were autologous. No placebo / control group used in the study. Paper did not answer the PICO. Lameness levels not measured. Review papers.
Inclusion:	Allogenic stem cells compared to a placebo / control group. Any allogenic stem cell type. Clinical signs of osteoarthritis visible for 1 month or greater. Answered the PICO. Measured lameness levels. Paper written in the English language.

**Search Outcome:** 

Database	Number of results	Excluded – Paper did not answer the PICO	Excluded – Study group did not compare to placebo / control	Excluded – Did not use purely allogenic canine stem cells	Excluded – Did not measure lameness as an outcome	Total relevant papers
CAB Abstracts	26	18	4	1	0	3
Scopus	33	24	2	3	1	3
PubMed	28	21	3	1	0	3
Total relevant papers when duplicates removed						3

## Conflict of Interest

The author declares no conflicts of interest.
